# Evaluating the sensitivity of droplet digital PCR for the quantification of SARS-CoV-2 in wastewater

**DOI:** 10.3389/fpubh.2023.1271594

**Published:** 2023-12-27

**Authors:** Magali de la Cruz Barron, David Kneis, Michael Geissler, Roger Dumke, Alexander Dalpke, Thomas U. Berendonk

**Affiliations:** ^1^Institute of Hydrobiology, Technische Universität Dresden, Dresden, Germany; ^2^Institute of Medical Microbiology and Virology, University Hospital Carl Gustav Carus, Technische Universität Dresden, Dresden, Germany; ^3^Department of Infectious Diseases, Medical Microbiology and Hygiene, University Heidelberg, Heidelberg, Germany

**Keywords:** droplet-digital PCR, RTqPCR, SARS-CoV-2, sensitivity, wastewater surveillance

## Abstract

Wastewater surveillance for SARS-CoV-2 has been demonstrated to be a valuable tool in monitoring community-level virus circulation and assessing new outbreaks. It may become a useful tool in the early detection and response to future pandemics, enabling public health authorities to implement timely interventions and mitigate the spread of infectious diseases with the fecal excretion of their agents. It also offers a chance for cost-effective surveillance. Reverse transcription-quantitative polymerase chain reaction (RTqPCR) is the most commonly used method for viral RNA detection in wastewater due to its sensitivity, reliability, and widespread availability. However, recent studies have indicated that reverse transcription droplet digital PCR (RTddPCR) has the potential to offer improved sensitivity and accuracy for quantifying SARS-CoV-2 RNA in wastewater samples. In this study, we compared the performance of RTqPCR and RTddPCR approaches for SARS-CoV-2 detection and quantification on wastewater samples collected during the third epidemic wave in Saxony, Germany, characterized by low-incidence infection periods. The determined limits of detection (LOD) and quantification (LOQ) were within the same order of magnitude, and no significant differences were observed between the PCR approaches with respect to the number of positive or quantifiable samples. Our results indicate that both RTqPCR and RTddPCR are highly sensitive methods for detecting SARS-CoV-2. Consequently, the actual gain in sensitivity associated with ddPCR lags behind theoretical expectations. Hence, the choice between the two PCR methods in further environmental surveillance programs is rather a matter of available resources and throughput requirements.

## 1 Introduction

As of 5th May 2023, the World Health Organization (WHO) chief declared the end to the severe acute respiratory syndrome coronavirus 2 (SARS-CoV-2) pandemic as a global health emergency (1). Nevertheless, the health threat of SARS-CoV-2 is still present, and the risk of future pandemics is increased (2).

New initiatives have been launched to improve further pandemic preparedness, including advancements in reliable and rapid detection methods, routine sample collection, data analysis, and data reporting protocols that are crucial to guide national and local strategies to mitigate the spread of pathogens with epidemic and pandemic potential (3).

Wastewater-based Epidemiology (WBE), also known as wastewater surveillance (4), has proven to be a valuable tool for tracking the prevalence of SARS-CoV-2 in the population, covering both symptomatic and asymptomatic cases, independent of the local test regime (5, 6). Still, WBE has its limitations, and special efforts need to be addressed to reduce methodological uncertainties through the implementation of standardized protocols, guidelines, and quality control measures that can ensure consistency and comparability of results across different regions and laboratories (7, 8). By focusing on accurate and sensitive detection methods and implementing robust data analysis and interpretation techniques, wastewater surveillance can become an effective tool for identifying new outbreaks of infectious diseases and enabling public health authorities to implement timely interventions during future pandemics.

Quantitative real-time polymerase chain reaction (qPCR), specifically reverse transcription quantitative real-time PCR (RTqPCR), has been the most commonly utilized benchmark technique for the detection of SARS-CoV-2 in clinical and non-clinical settings (7, 9). However, the precision and sensitivity of RTqPCR have been questioned, especially at low viral concentrations (10, 11).

Droplet digital PCR (ddPCR) is an emerging technology to quantify nucleic acids with high accuracy and sensitivity (12–14). It is characterized by the random partitioning of the target molecules across thousands of droplets in which PCR takes place. Poisson statistics allow quantifying the absolute number of target molecules in the sample to be inferred directly from the number of positive and negative droplets (15, 16). This is in contrast to RTqPCR, which requires that the measured signals align with a calibration curve constructed from serially diluted standards.

Several clinical studies suggest that reverse transcription droplet digital PCR (RTddPCR) outperforms RTqPCR for the detection and quantification of SARS-CoV-2, offering higher sensitivity, better accuracy, and lower susceptibility to PCR inhibitors (9, 17–20). However, studies comparing the performance of both PCR methods on clinical samples often report inconclusive data that do not provide enough or clear evidence to support one of the methods as superior to the other, making a direct comparison difficult. These methodic inconsistencies include RTqPCR efficiencies out of the acceptable range (Minimum information for publication of quantitative real-time PCR experiments guidelines, MIQE, guidelines) (9, 18), different methods for determination of the limits of detection (LOD), and limits of quantification (LOQ) (17, 19), and the use of different primer/probe sets (17, 19).

Fewer studies report the applicability and performance of RTddPCR for the detection of SARS-CoV-2 in wastewater with conflicting or inconclusive results (21–24). For example, Ahmed et al. (21) that RTddPCR was 2–5 times more sensitive than RTqPCR using the CDC N1 and N2 assays for SARS-CoV-2 detection in wastewater. In contrast to those results, D’Aoust et al. (23) observed higher sensitivity (2.5 times more) when using RTqPCR than RTddPCR for the same CDC assays when wastewater solids were analyzed. However, the latter study does not provide sufficient information on how the LODs were determined. Consequently, further research should corroborate these results and verify the possible advantages or disadvantages of RTddPCR over RTqPCR in the context of wastewater surveillance.

In this study, we tested and compared the performance of RTddPCR and RTqPCR for detecting and quantifying SARS-CoV-2 in 59 wastewater samples collected across six wastewater treatment plants in Saxony, Germany. The analyzed samples were collected during the third epidemic wave, a phase marked by periods of low SARS-CoV-2 incidence, allowing the assessment of the sensitivity detection of both PCR approaches and the extent of variability in samples with low viral concentrations. Given the importance of having accurate and reproducible WBE detection methods for future pandemics, by carrying out a side-by-side comparison, our work evaluates the strengths and limitations of RTddPCR in wastewater surveillance and its usefulness as an alternative tool to overcome the sensitivity limitations of RTqPCR for SARS-CoV-2 measurements.

## 2 Materials and methods

### 2.1 Samples

A total of 59 composite (24-h) raw wastewater samples were collected from May 2021 to July 2021 at six wastewater treatment plants in Saxony, southeastern Germany (Supplementary Table S1) as described in Helm et al. (25). The sampling period involved the main phase of the third epidemic wave (May–June 2021) in Saxony, characterized by a low incidence period (25). Samples were collected two to seven times per week, stored at 4°C, and transported to the laboratory for analysis within three days.

### 2.2 Virus concentration and RNA extraction

Viruses in environmental samples were concentrated by polyethylene glycol precipitation (PEG) as described by Dumke et al. (26). Briefly, 45 mL of raw wastewater was mixed with 10% (w/v) PEG 8000 and 2.25% NaCl and centrifuged at 4°C and 12,000 × *g* for 1.5 h. The obtained pellet was resuspended in 500 μL sterile PBS (pH 7.4). 200 μL were used for total RNA extraction, using the RNeasy kit (Qiagen, Hilden, Germany) following the manufacturer’s instructions. PCR inhibitors were removed from the 50 μL of eluted RNA, using the OneStep PCR Inhibitor Removal Kit (Zymo Research, Irvine, CA, United States). All RNAs were stored at −80°C until use for RTqPCR and RTddPCR analysis.

### 2.3 Quantification of the *E* gene by RTqPCR and RTddPCR

For a direct comparison between PCR platforms, the quantification of the *E* gene of SARS CoV-2 in RNA extracted from wastewater samples was carried out by one-step RTqPCR and One-Step RT-ddPCR assays using the same primers and probe listed in Table 1.

**Table 1:** Primers and probe used in this study.

Primer name	Sequence (5′ > 3′)	Target gene	Product size (bp)	Reference
E_Sarbeco_F	ACAGGTACGTTAATAGTTAATAGCGT	*E* gene	113	Corman et al. (27)
E_Sarbeco_R	ATATTGCAGCAGTACGCACACA			
E_Sarbeco_P1	[FAM]ACACTAGCCATCCTTACTGCGCTTCG[BBQ650]			

One-step RTqPCR reactions comprised 10 μL of Luna Universal Probe One-Step Reaction Mix (2X, NEB, Ipswich, MA, United States), 4 μL of RNA, 400 nM each of forward and reverse primers along with 250 nM of the probe in a final reaction volume of 20 μL. All samples were tested in duplicates in a Bio-Rad CFX96 thermal cycler (Bio-Rad Laboratories, Richmond, CA). The PCR program, previously optimized for primers and probe concentration and annealing temperature, was set as follows: one cycle at 55°C for 5 min, 95°C for 1 min followed by 45 cycles at 95°C for 10 s, and 61°C for 60 s. The concentration of the *E* gene was calculated from standard curves generated with 6-point serial dilutions (10-fold) of a synthetic SARS-CoV-2 RNA control (Wuhan strain, Twist Bioscience, San Francisco, CA, United States) at a stock concentration of 2.2 × 10^5^ copies/μL, previously determined by ddPCR. Samples were deemed positive for SARS-CoV-2 detection if amplification occurred in at least one of the two replicates within 45 cycles (28). Samples were considered quantifiable if concentrations exceeded the limit of quantification (LOQ).

One-Step RT-ddPCR assays, reactions contained 5 μL of supermix One-step RT-ddPCR Advanced Kit for Probes (Bio-Rad), 2 μL reverse transcriptase, 1 μL of 300 mM dithiothreitol, 4 μL of RNA, 900 nM each of forward and reverse primers along with 250 nM of the probe (Table 1) in a final reaction volume of 20 μL. Samples were tested in technical duplicates. Each 96-well PCR plate analyzed included positive and negative (EDX SARS-CoV-2 positive and negative Run Controls, BioRad) and two non-template controls. Droplets were generated on a QX200 droplet generator (Bio-Rad) in accordance with the manufacturer’s instructions and then transferred into a 96-well PCR plate (heat-sealed with a foil plate seal, Bio-Rad). PCR was carried out in a C1000 thermal cycler (Bio-Rad) with cycling conditions set as follows: one cycle at 50°C for 60 min, 95°C for 10 min followed by 45 cycles at 94°C for 30 s, 61°C for 60 s, and a final cycle at 98°C for 10 min, and hold at 4°C until droplet reading. Ramp rate was set to 2.0°C/s. The droplets were read using a QX200 droplet reader and data was subsequently analyzed using QuantaSoft Software v1.4.0.99 (Bio-Rad). The threshold for distinguishing between negative and positive droplets was set manually just above the cluster of negative droplets. The absolute abundances were calculated by QuantaSoft Software, based on the Poisson distribution and the number of positive and negative droplets of the target gene (14). Samples were considered positive if at least two positive droplets were detected in at least one of the two replicates, and the number of accepted droplets was >10,000 per sample well (29). Samples were considered quantifiable if the concentrations were above the LOQ.

### 2.4 Limits of detection (LOD) and quantification (LOQ)

To determine the LOD, we started with two samples of the synthetic SARS-CoV-2 RNA control at known concentrations of 2.0 × 10^4^ and 5.0 × 10^5^ copies/μL, respectively. From each of the two samples, we prepared 10-fold dilutions covering at least six orders of magnitude until we reached concentrations as low as 0.2 and 0.5 copies/μL, respectively. The dilution series were augmented by intermediate dilutions (using factors of 2 and 4) to improve the resolution at concentrations below 50 copies/μL. For each dilution, eight replicate measurements were performed with both RTqPCR and RTddPCR as described in section 2.3. The results were used to fit a binomial regression model *f ~ c* relating the fraction of positive outcomes (f; range 0–1) to concentration (c). The LOD was set at *f* = 0.95 and thus represents the lowest concentration associated with false negative rates below 5%.

The LOQ was identified from the same set of measurements used for the calculation of LOD. In this case, we fitted an exponential model cv. *~ c* relating the coefficient of variation in replicate measurements (*cv*) to concentration. The LOQ was defined as the concentration where *cv.* < 0.25. Thus, the LOQ represents the concentration where the standard deviation among replicates does not exceed 1/4 of the observed mean.

Uncertainties in the estimates of LOD and LOQ were expressed as 95% confidence intervals. The latter were obtained by a bootstrap approach (R package “boot”) which treats the distinct concentration levels (dilutions) as strata.

### 2.5 Inhibition test

Ten RNA extracts from wastewater samples that did not result in amplification by both RTqPCR and RTddPCR were pooled and used as a diluent. PCR inhibition was tested on a tenfold serial dilution series (from 10^3^ to 1 gene copies/20 μL reaction) of synthetic SARS-CoV-2 RNA control prepared on RNA extracts from wastewater samples and compared with values obtained when prepared on DNase- and RNase-free water. At each step in the dilution series, three replicates were tested for both RTqPCR and RTddPCR as described in section 2.3. The presence of PCR inhibition in samples was evaluated as described by Ahmed et al. (28). Briefly, for RTqPCR, if the Cq value of the samples when diluted on RNA extracts was >2 Cq values compared to the Cq value for PCR grade water, the sample was classified as containing PCR inhibitors. Regarding RTddPCR, if the concentrations of samples, when diluted in RNA extracts, were four times lower than those diluted in PCR-grade water, the sample was deemed to be inhibited.

## 3 Results

### 3.1 Comparison of RTddPCR and RTqPCR performance on benchmark samples

To compare the sensitivity and assess differences attributed to the PCR platforms themselves, the same set of primers and probes were used, templates and RNA volumes were identical, and reactions were run simultaneously, such that variability due to sample variations or reagents was reduced to the minimum. PCR assays (primer concentration (RTqPCR), annealing temperature, and cycling number) were optimized for each PCR approach before comparison. A thermal gradient was used for optimizing the annealing temperature of RTddPCR assays (Supplementary Figure S1). The mean amplification efficiency for optimized RTqPCR assays was 101.45% ± 2.44% (95% CI), the mean correlation coefficient (*r*^2^) was 0.997 ± 0.002 (95% CI), the mean slope and *y*-intercept values were − 3.29 ± 0.0663 (95% CI) and 39.80175 ± 0.227 (95% CI), respectively.

**Figure 1:**
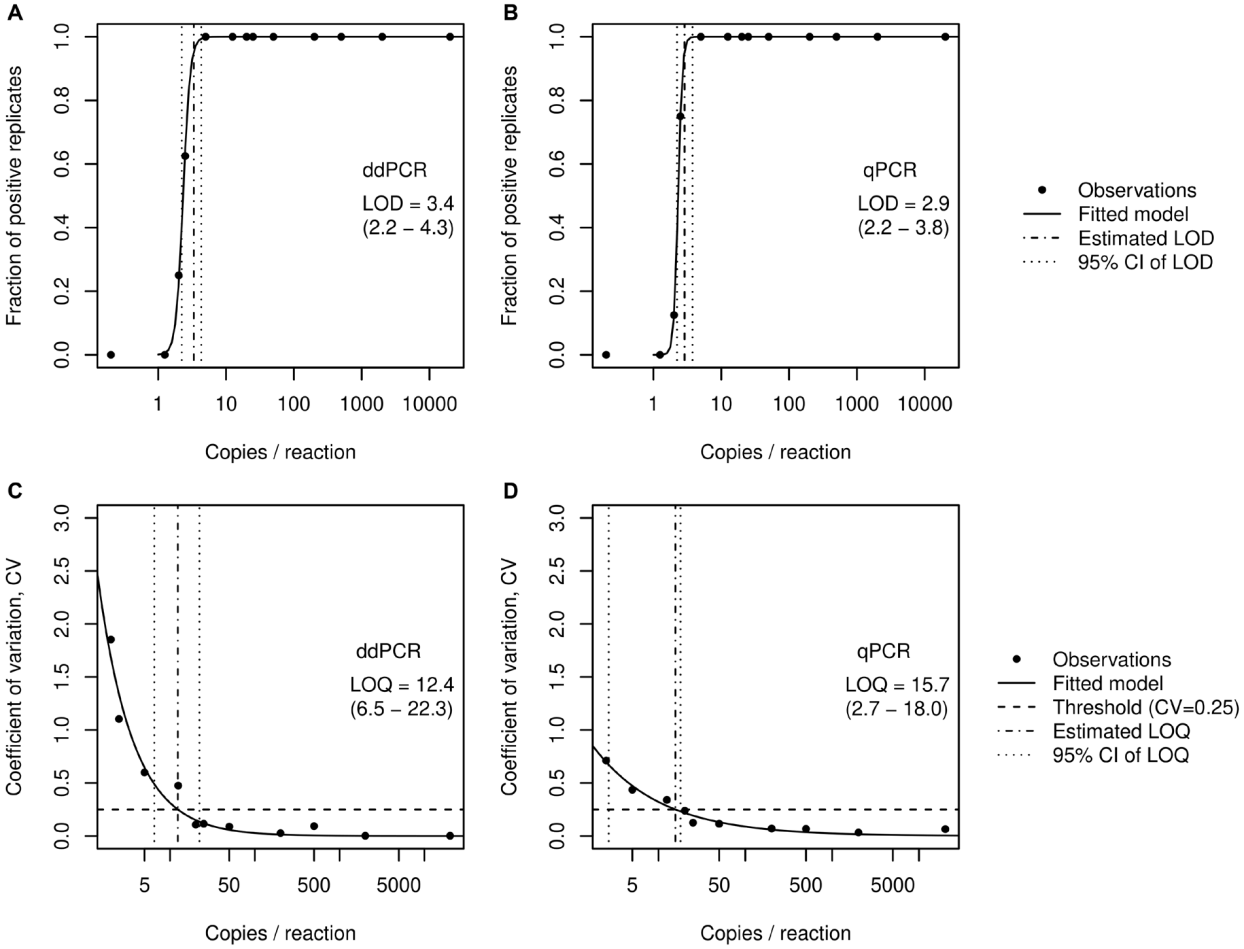
Determination of the limits of detection (LOD) and limits of quantification (LOQ) for the *E* gene of SARS-CoV-2 by RTddPCR **(A,C)** and RTqPCR **(B,D)**. Dots represent the fraction of positive cases and the empirical coefficient of variation, respectively, each computed from eight replicates per concentration. Solid lines represent the fitted logistic models **(A,B)** or exponential models **(C,D)** used to find LOD and LOQ.

Both RTddPCR and RTqPCR robustly quantify input RNA across the tested range and exhibit similar LODs with regard to the *E* gene of SARS-CoV-2 at 3.4 (2.2–4.3) and 2.9 (2.2–3.8) gene copies/ 20 μL reaction, respectively (Figure 1; numbers in parentheses represent 95% CI). This corresponds to 2.4 × 10^3^ and 2.1 × 10^3^ gene copies per liter of wastewater, for RTddPCR and RTqPCR, respectively. The LOQs were determined at 12.4 (6.5–22.3) and 15.7 (2.7–18.0) gene copies/ 20 μL reaction, corresponding to 8.7 × 10^3^ and 1.1 × 10^4^ gene copies per liter of wastewater for RTddPCR and RTqPCR, respectively.

### 3.2 Quantification of SARS-CoV-2 in wastewater samples with low viral load

Fifty-nine samples were collected from May to July 2021 to study the performance of RTqPCR and RTddPCR on samples with particularly low SARS-CoV-2 concentrations. No inhibition was identified in the tested samples when using RTqPCR or RTddPCR assays. No amplification was observed in any negative control of either method.

**Figure 2:**
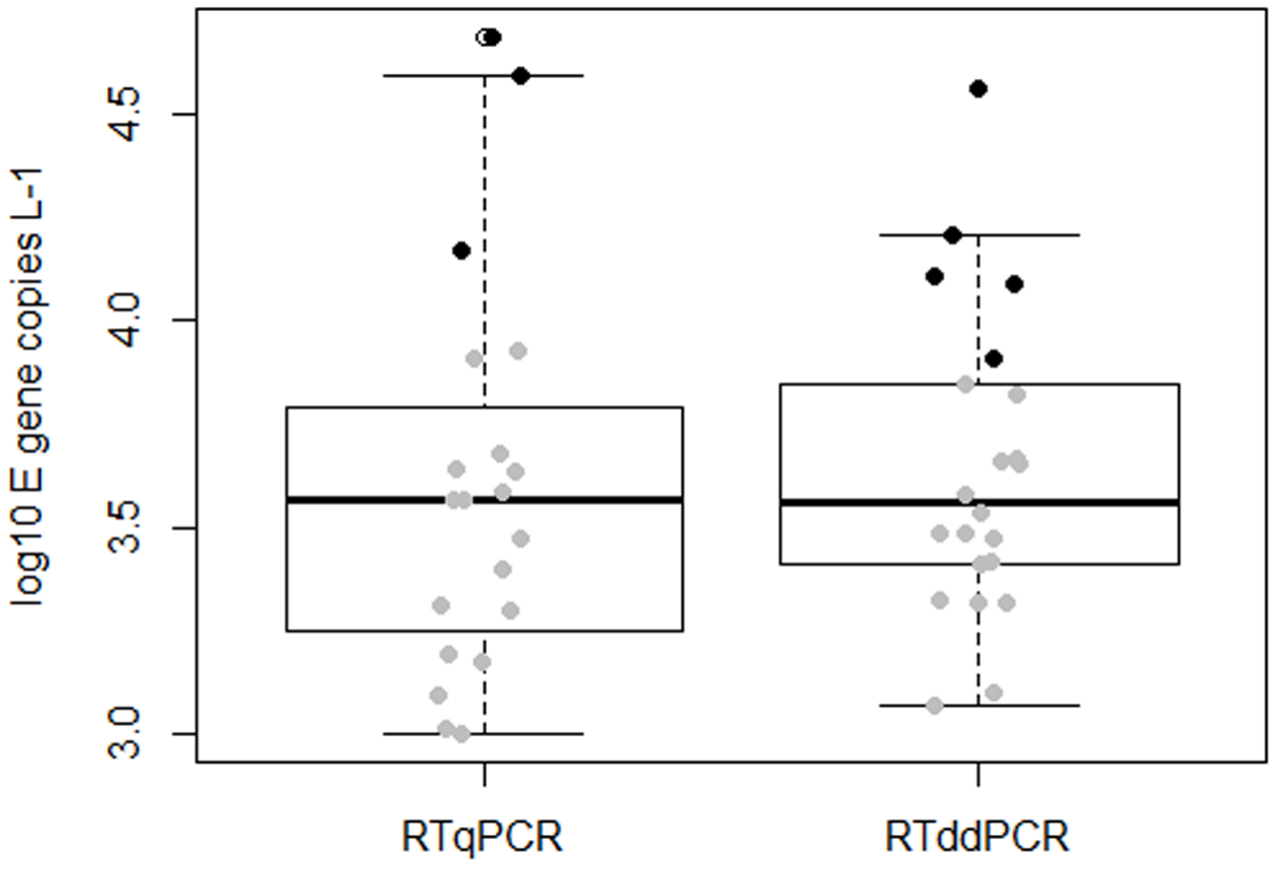
Quantitative detection of SARS-CoV-2 in wastewater samples by RTqPCR and RTddPCR. Filled dots in the boxplot represent individual measurements with gray color indicating values below the LOQs of 1.1 × 10^4^ and 8.7 × 10^3^ gene copies per liter of wastewater for RTqPCR and RTddPCR, respectively, and black color indicating values above the respective LOQs.

Both RTqPCR and RTddPCR measurements indicated similar concentrations of SARS-CoV-2 in wastewater. Out of the 59 samples analyzed, 20 (34%) showed positive amplification in RTqPCR and 22 (37%) in RTddPCR (Figure 2). Considering the limits of detection identified for the individual PCR platforms (Section 3.1), the number of samples to be actually regarded as positive reduces to 14 for the RTqPCR approach and 17 for RTddPCR (Figure 2, grey dots). The number of samples with measured concentrations above the respective limits of quantification (LOQ, section 3.1) amounts to three and five for RTqPCR and RTddPCR, respectively (Figure 2, black dots). In any case, the slightly higher proportion of either positive, detectable, or quantifiable samples obtained for RTddPCR in comparison to RTqPCR was not statistically significant (Fisher’s exact test; positive amplification: *p* = 0.85; cases with concentrations > LOD: *p* = 0.68, cases with concentration > LOQ: *p* = 0.72). Likewise, a comparison of the concentrations obtained by the alternative technologies did not indicate a statistically significant shift in location (*p* = 0.5, Wilcoxon rank sum test). The observed mean values of the log10 concentrations and their corresponding standard errors (in parenthesis) were 3.60 (0.11) for RTqPCR and 3.64 (0.079) for RTddPCR, respectively. A formal power analysis based on the given sample sizes and observed standard deviations suggests that the study design would have allowed for the detection of a shift in location by 0.55 log units with both type I and type II error probabilities being confined to 5%. It is thus unlikely that a major difference in performance between the two methods was overlooked because of undersampling.

## 4 Discussion

Several studies have confirmed that SARS-CoV-2 wastewater surveillance correlates with trends observed in clinical surveillance [(29, 30); Supplementary Figure S2], and has been used to predict upcoming SARS-CoV-2 waves (30, 31). To maximize the benefits of wastewater surveillance in future pandemics, it is crucial to develop and employ accurate pathogen detection methods (32). In particular, wastewater detection methods must be sensitive enough to avoid potential underestimation in the case of low infection rates or sporadic shedding that may go undetected. In recent years, droplet digital PCR has emerged as a particularly promising technology in terms of sensitivity (33, 34).

Existing comparative studies suggest that ddPCR offers a higher sensitivity and precision than traditional RTqPCR for the detection and quantification of SARS-CoV-2 (9, 18, 21, 22). However, a fair comparison of the two approaches requires that identical primer-probe combinations are used and the effort put in methodological optimization is otherwise equal. Here, we demonstrated that, after proper optimization, the remaining difference in sensitivity between RTqPCR and RTddPCR is actually marginal (Figure 1). In particular, if the estimated LODs (or their confidence limits) are reasonably rounded to integer copies, the performance of the two methods appears to be indistinguishable. The determined LODs are relatively low in comparison with LODs reported for E_Sarbeco assays (28, 35), however, it should be noted that the experimental protocols and statistical methods employed to estimate the LODs differ from one study to another. Thus, for simple sample matrices, there appears to be no obvious reason to favor either of the two methods.

We wonder if the same level of sensitivity could be achieved when analyzing complex environmental matrices. Wastewater samples may contain inhibitory substances that can affect the PCR performance. While the majority of the studies indicate that RTddPCR is less susceptible to inhibition (7, 21), D’Aoust et al. (23) reported that RTddPCR was more inhibited than RTqPCR when wastewater influent solids were analyzed. In our study, the absence of inhibition in the samples analyzed should be interpreted with care as PCR inhibitors were largely removed from the extracted environmental RNAs by an additional sample treatment step. Our results demonstrate that inhibition of either PCR approach can be avoided if samples are cleaned up from inhibitory substances such as polyphenolic compounds, humic/fulvic acids, and related compounds prior PCR. Following such treatment, both RTqPCR and RTddPCR appear to be suitable for detecting and quantifying SARS-CoV-2 in complex matrices like wastewater. Overall, RTqPCR and RTddPCR yield similar positive SARS-CoV-2 detections in complex matrices like wastewater, even at low SARS-CoV-2 concentrations (Figure 2).

Our findings contradict the theoretical assumption that RTddPCR provides improved sensitivity over RTqPCR, particularly for low-abundance targets (Figures 1, 2). Therefore, the choice between qPCR and ddPCR for further environmental surveillance programs should be based on a careful evaluation of the specific needs, resources, and priorities of the study. Here are some considerations:

(a) *Relative quantification* vs. *absolute quantification:* qPCR and RTqPCR rely on cycle threshold (Ct) values to provide relative quantifications when controls are used as reference material and on calibration curves for quantification (36). However, several factors can influence Cq values, making them potentially misleading when used as the sole indicator of virus concentration (20, 37). ddPCR and RTddPCR count the number of positive and negative droplets to determine the absolute concentration of the target nucleic acid (34). This eliminates the variability associated with standard curve-based quantification methods used in qPCR, contributing to its reputation as a highly reproducible and precise quantification method.

According to our experience, the independence of reaction efficiency and the exclusion of controls for standard curve generation in ddPCR simplify assay development and reduce the effort required for optimization. Further, it provides an accurate and straightforward quantification process.

(b) *Workflow:* depending on the ddPCR platform employed, ddPCR workflow can be time-consuming and potentially tedious. For this study, we used a water–oil emulsion droplet-based ddPCR platform, for which RTddPCR experiments required more hands-on time (full 96 well plate preparation ~3 h, plus ~3 h PCR amplification program, plus ~2 h droplet reader, to determine which contain a target and which do not) than RTqPCR (full 96 well plate preparation ~1.5 h, plus ~1.5 h PCR amplification program/ fluorescence detection). To streamline time-consuming workflows in ddPCR alternative ddPCR platforms, such as microfluidic chip-based systems, could be used. These advanced platforms offer a significant reduction in hands-on time and sample processing steps (38).

(c) *Throughput requirements*: based on point (b), the use of ddPCR can significantly limit the throughput for large-scale wastewater surveillance initiatives. However, using ddPCR as a confirmatory tool can enhance confidence in qPCR results, especially for critical data or when facing unexpected or conflicting findings.

(d) *Resource availability:* RTqPCR is more commonly available and established in many laboratories, making it a readily accessible option (39). On the other hand, ddPCR might require a higher initial investment in equipment, and reagents and consumables are more expensive than RTqPCR (22). However, improperly optimized qPCR protocols can result in dramatically increased costs.

In addition, the variability introduced by human errors may make it difficult to directly compare the two methods, especially in terms of accuracy and precision. Errors in pipetting, contamination, and sample handling can create discrepancies between the two techniques. To minimize the effect of human errors, rigorous adherence to best practices, quality control measures, and validation experiments are essential.

Finally, the choice of the target gene might directly influence the sensitivity of the detection method (40, 41). In this study, our focus has been exclusively on the E gene. Therefore, future studies should consider incorporating alternative target genes into their sensitivity assessments to enhance the reliability and applicability of wastewater surveillance methods in monitoring the prevalence of SARS-CoV-2 and its variants in the community.

In this study, we thoroughly compared the performances of the two alternatives, RTqPCR and RTddPCR, for the detection and quantification of SARS-CoV-2 in wastewater. Our results indicate that, if conventional RTqPCR is properly optimized, the possible gain in sensitivity and precision attainable through the use of RTddPCR is marginal. Acknowledging the similar performance of RTqPCR and RTddPCR has important practical consequences: It allows researchers and practitioners to choose the method that best suits their resources, laboratory facilities, the skills of personnel, and the specific demands of the PCR application. This is especially important in resource-constrained settings where access to particular equipment might be limited.

Furthermore, the comparable performance of these two PCR-based methods has also implications for the robustness of WBE. Having two equally sensitive methods at hand means that, in case of challenges (e.g., interference from substances in wastewater), one method can serve as a complement or substitute for the other. However, independently of the chosen PCR approach, it is essential to minimize assay variability to promote data comparability and facilitate the establishment of robust WBE monitoring systems that effectively contribute to public health and pandemic management.

## Data availability statement

The raw data supporting the conclusions of this article will be made available by the authors, without undue reservation.

## Author contributions

MB: Conceptualization, Data curation, Formal analysis, Investigation, Methodology, Validation, Visualization, Writing – original draft, Writing – review & editing. DK: Conceptualization, Data curation, Formal analysis, Investigation, Methodology, Validation, Visualization, Writing – review & editing. MG: Conceptualization, Investigation, Methodology, Validation, Writing – review & editing. RD: Conceptualization, Formal analysis, Funding acquisition, Investigation, Methodology, Writing – review & editing. AD: Conceptualization, Formal analysis, Funding acquisition, Investigation, Writing – review & editing. TB: Conceptualization, Formal analysis, Funding acquisition, Investigation, Project administration, Visualization, Writing – review & editing.
