# Supplementary material for: Evaluating the sensitivity of droplet digital PCR for the quantification of SARS-CoV-2 in wastewater
Source: Front Public Health. 2023 Dec 27;11:1271594. doi: 10.3389/fpubh.2023.1271594 (PMC10903512; doi:10.3389/fpubh.2023.1271594)
Supplement: Supplementary file 1 [file Data_Sheet_1.docx]

Supplementary Material

# Supplementary Figures and Tables

## Supplementary Figures


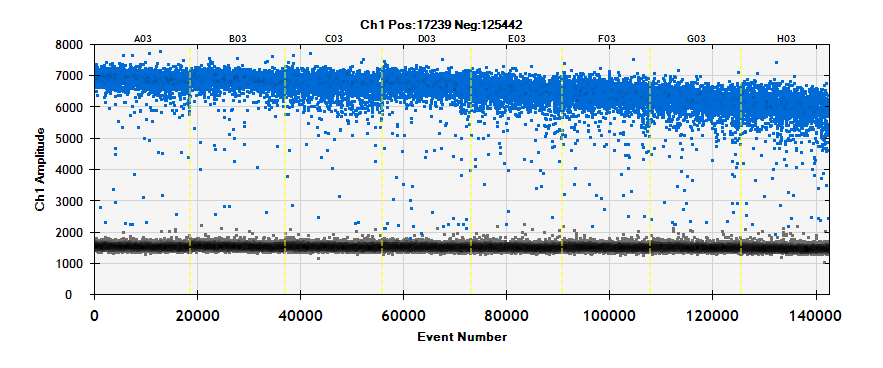


**Supplementary Figure 1.** Thermal gradient PCR for optimizing annealing temperature. Applied temperatures ranged from 62°C (A03) to 54°C (H03) and were tested on synthetic RNA. We determined 61°C (B03) was an optimal annealing temperature for the experiment which resulted in clear band separation and high fluorescence amplitude.


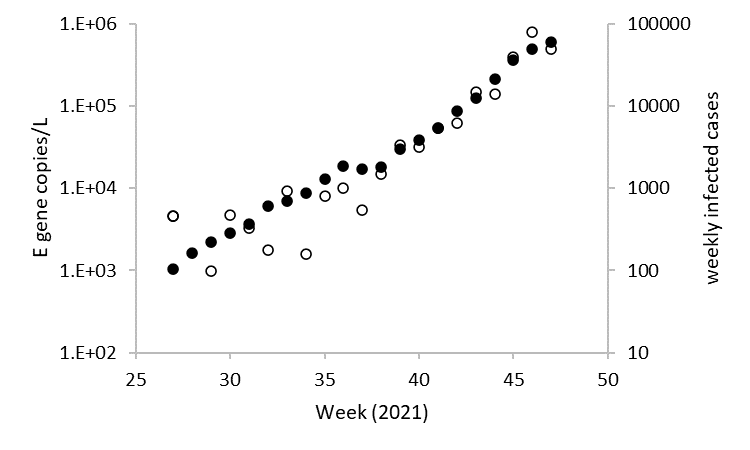


**Supplementary Figure 2.**  SARS-CoV-2 concentration in raw wastewater and the prevalence of the virus in Saxony, Germany, from July (week 27) to November (week 47) 2021. The concentration of SARS-CoV-2 E gene was determined by ddPCR in wastewater samples (n=145) collected from six wastewater facilities in 2021 (Empty circles). The weekly number of confirmed cumulative clinical SARS-CoV-2 cases at the state level (filled black circles) was obtained from the official statistics disseminated by the responsible federal authority (Robert Koch Institute [(42)](https://www.zotero.org/google-docs/?nJkWoh), [RKI - Coronavirus SARS-CoV-2 - Wochenberichte zu COVID-19 (bis 8.6.2023). [cited 2023 Oct 20]. Available from: https://www.rki.de/DE/Content/InfAZ/N/Neuartiges_Coronavirus/Situationsberichte/Wochenbericht/Wochenberichte_Tab.html?nn=13490888](https://www.zotero.org/google-docs/?mYQkGB))

## Supplementary Tables

#### Supplementary Table 1. Characteristics of sampled wastewater treatment plants located in Saxony, Germany [(25)](https://www.zotero.org/google-docs/?Ni1eIe).

| **Wastewater treatment plant** | **Connected inhabitants**  **[x1,000 persons]** | **Number of connected municipalities** | **Combined / separate sewer [%]** |
| --- | --- | --- | --- |
| Morgenröthe | 2.4 | 2 | 30 / 70 |
| Elsterberg | 2.7 | 1 | 95 / 5 |
| Schönfeld | 29.4 | 5 | 85 / 15 |
| Plauen | 68.7 | 1 | 97 / 3 |
| Chemnitz | 251 | 3 | 62 / 38 |
| Dresden | 702 | 13 | 75 / 25 |
